# A New Look into the Mode of Action of Metal-Based Anticancer Drugs

**DOI:** 10.3390/molecules25020246

**Published:** 2020-01-07

**Authors:** M. Paula M. Marques, Ana L. M. Batista de Carvalho, Adriana P. Mamede, Asha Dopplapudi, Svemir Rudić, Madhusudan Tyagi, Victoria Garcia Sakai, Luís A. E. Batista de Carvalho

**Affiliations:** 1 Unidade de I&D Química-Física Molecular, Department of Chemistry, University of Coimbra, 3004-535 Coimbra, Portugal; pmc@ci.uc.pt (M.P.M.M.); apm@uc.pt (A.P.M.); labc@ci.uc.pt (L.A.E.B.d.C.); 2 Department of Life Sciences, University of Coimbra, 3000-456 Coimbra, Portugal; 3 ISIS Facility, STFC Rutherford Appleton Laboratory, Chilton, Didcot OX11 0QX, UK; asha.dopplapudi@stfc.ac.uk (A.D.); svemir.rudic@stfc.ac.uk (S.R.); victoria.garcia-sakai@stfc.ac.uk (V.G.S.); 4 NIST Center for Neutron Research, Gaithersburg, MD 20899, USA; madhusudan.tyagi@nist.gov; 5 Department of Materials Science and Engineering, University of Maryland, College Park, MD 20742, USA

**Keywords:** palladium anticancer drug, DNA, hydration water, neutron scattering techniques, Raman, FTIR

## Abstract

The mode of action of Pt- and Pd-based anticancer agents (cisplatin and Pd_2_Spm) was studied by characterising their impact on DNA. Changes in conformation and mobility at the molecular level in hydrated DNA were analysed by quasi-elastic and inelastic neutron scattering techniques (QENS and INS), coupled to Fourier transform infrared (FTIR) and microRaman spectroscopies. Although INS, FTIR and Raman revealed drug-triggered changes in the phosphate groups and the double helix base pairing, QENS allowed access to the nanosecond motions of the biomolecule’s backbone and confined hydration water within the minor groove. Distinct effects were observed for cisplatin and Pd_2_Spm, the former having a predominant effect on DNA’s spine of hydration, whereas the latter had a higher influence on the backbone dynamics. This is an innovative way of tackling a drug’s mode of action, mediated by the hydration waters within its pharmacological target (DNA).

## 1. Introduction

Cancer is a growing health problem, currently the second-leading cause of death worldwide (9.6 million deaths in 2018). New and more efficient chemotherapeutic strategies are therefore an urgent clinical need. Over the years numerous cytostatics have been developed, aiming at an improved antineoplastic activity coupled to decreased deleterious side effects. Platinum drugs were introduced in the late 1960s [1,2], upon the serendipitous discovery of cisplatin (cis-(NH_3_)_2_PtCl_2_). However, there are serious limitations to the clinical use of these types of drugs, namely severe side effects (e.g., nephrotoxicity, hepatotoxicity or myelosuppression) and acquired resistance [3,4,5]. Additionally, they have shown poor efficacy against metastatic cancers, which are responsible for higher mortality worldwide and therefore need improved oncology therapies. A promising alternative comes from metal-based agents comprising more than one metal centre, such as Pt(II) and Pd(II) polynuclear chelates with polyamines, that constitute a specific class of DNA-damaging anticancer compounds displaying a non-conventional mechanism of action that may lead to an enhanced therapeutic effect. Cytotoxicity arises from the selective covalent binding of the metal ions to DNA purine bases at more than one site within the double helix, yielding long-range interstrand adducts. These agents have been synthesised and extensively investigated by the team, to provide a comprehensive set of data on (i) cytotoxicity towards several human cancer cells [6,7,8,9,10], (ii) interactions with DNA and glutathione-mediated resistance pathways [11,12] and (iii) impact on cellular metabolism and intracellular water [12,13,14,15,16]. Vibrational spectroscopy techniques were applied (including inelastic neutron scattering and synchrotron radiation-Fourier transform infrared (FTIR)) coupled to theoretical simulations [4,17,18], as well as synchrotron-based Extended X-ray Absorption Fine Structure (EXAFS) [11]. The dinuclear complex Pd_2_Spm (Spm = spermine, H_2_N(CH_2_)_3_NH(CH_2_)_4_NH(CH_2_)_3_NH_2_), in particular, has yielded quite promising results towards human triple negative (metastatic) breast cancer, coupled to lower toxicity in non-tumorigenic cells [8,10].

As suggested in previous studies by the authors [12,15,16,19], apart from the conventional targets of these type of metal-based agents (DNA and specific proteins), there may be other receptors that are relevant for chemotherapeutic activity, namely water molecules—both within the cytoplasm and the hydration layers of biomolecules. Intracellular water (cytoplasmic), which accounts for 70 to 80% of the whole cell mass, has a particular structural and dynamical behaviour namely its ability to form highly organised H-bond networks. It underlies vital biological activities, from the maintenance of the stable and functional three-dimensional architecture of biopolymers to its direct role in essential biochemical processes such as protein folding, enzyme catalysis and intracellular transport [20,21,22,23,24,25,26,27]. In turn, water in the vicinity of biomolecules (hydration layers) has properties that are noticeably different from cytoplasmic and bulk water, and can be viewed as a defect in the regular H-bond arrangement of the water molecules within the cellular milieu, induced by the biopolymer’s surface which is chemically and topologically inhomogeneous and prone to conformational rearrangements. Hydration water is crucial for bioactivity, the first hydration shell being an essential part of a biomolecule’s structure that regulates its structural preferences and physiological role [23,26,28,29]. Therefore, any disruption of a biopolymer’s hydration sheath may affect its conformational and dynamical profiles, with consequences on biofunctionality which are still misunderstood. Likewise, the influence of a drug on the water layer in the vicinity of biomolecules (e.g., proteins, RNA or DNA) is an unexplored issue. These perturbations are not identical everywhere, the effect on some sites being more significant than on others (e.g., hydrophobic groups, H-bond donors or acceptors, outer hydration shell or inner hydration pockets).

Neutron spectroscopic techniques, namely inelastic and quasielastic neutron scattering (INS and QENS), are particularly suited for probing structure and dynamics of water in its various forms, including interfacial water in biological matrices, at nano- to picosecond timescales and on atomic lengthscales. INS allows the authors to define the local structure of water molecules within the hydration shells of biomolecules [30,31,32,33,34] as well as to directly access the whole set of vibrational modes, with high sensitivity to both the intra- and intermolecular modes, and yield complementary information to that obtained by optical vibrational spectroscopy (infrared and Raman). Translational modes of water are changed near the biopolymers and appear in a different energy transfer range to the vibrations from the biomolecule itself, thus rendering INS a very useful tool to probe water. QENS, in turn, is a method of choice for directly accessing different spatially resolved dynamical processes of key biological components—from fast localised modes to slower global translations—and the way they are changed by the presence of a drug, [12,15,16,19,26,35,36,37,38,39,40,41,42,43]. While QENS is a direct probe of water’s dynamical behaviour, INS and the optical vibrational techniques Raman and FTIR indirectly monitor water properties which affect the corresponding vibrational profile while detecting conformational rearrangements of the biomolecule.

Following a successful QENS study that revealed changes induced by the drug to the dynamical processes, occurring at the picosecond timescale, within DNA’s hydration shell [18], the current work focuses on the effects on the slower nanosecond motions of the nucleic acid skeleton (backbone) and the biopolymer’s constrained hydration waters. The use of a high resolution QENS instrument such as the high flux backscattering spectrometer (HFBS) at the National Institute of Standards and Technology Centre for Neutron Research (NCNR, Gaithersburg, MD, USA), enabled the detection of this type of dynamical processes, thus allowing to build a complete dynamical picture of the drug’s impact on DNA.

This study was carried out for both H_2_O- and D_2_O-hydrated DNA (B-DNA, r.h. >80%) with and without the drug (Pd_2_Spm and cisplatin), as well as for lyophilised DNA. INS, QENS and complementary optical vibrational spectroscopy techniques—FTIR in attenuated total reflectance mode (FTIR-ATR) and Raman microspectroscopy (microRaman)—were applied, for gathering accurate information on the drug’s impact on DNA’s structural and dynamical profiles.

## 2. Materials and Methods

The list of chemicals and the experimental description regarding the synthesis and characterisation of the Pd_2_Spm complex are described in the Supporting Information, together with details of the INS, QENS, FTIR-ATR and microRaman data acquisition and analysis.

### 2.1. Preparation of Drug-DNA Samples

Hydrated DNA samples were analysed, both untreated and drug-treated—with Pd_2_Spm and the clinically used mononuclear Pt-drug cisplatin (as a reference). The drug concentration (8 μM) was chosen according to the IC_50_ values (drug dosage leading to 50% cell death) previously measured for cisplatin and Pd_2_Spm in distinct human cancer cell lines [8,14]. In addition, dry DNA samples, in powder form and without drug, were also probed, lyophilised immediately before the measurements, hereafter represented by DNA_lyoph_.

Fibrous commercial DNA (from calf-thymus) was used to prepare the samples without and with drug, the latter made by solubilising 250 mg of DNA fibres in 125 mL of either cisplatin or Pd_2_Spm solution at 8 μM with gentle shake for ~24 h (at 4 °C). Aqueous drug solutions were used (instead of saline solutions) in order to ensure a prompt hydrolysis of the chlorides, which is essential for drug activation prior to DNA binding. 1/10 volumes of 3 M-sodium acetate and 3 volumes of ethanol (≥99.8%) were then added, followed by a 2 h incubation (at −20 °C). The solutions were centrifuged at 4075× *g* for 20 min (4 °C), the pellets were washed twice with ethanol (70%) and centrifuged again (under the same conditions).

H_2_O- and D_2_O-hydrated DNA were prepared at a r.h. >80%, to ensure the stability of the native B conformation, with a complete primary hydration shell, yielding the samples now denoted as H_2_O-DNA_hyd_ and D_2_O-DNA_hyd_, respectively. This was achieved following a previously reported procedure [12]: the DNA was placed in a desiccator (closed environment) with a saturated KCl solution (in either H_2_O or D_2_O) until attaining a stable weight (corresponding to a r.h. of 83.62–84.34%, at 25 °C).

### 2.2. FTIR-ATR

The FTIR-ATR spectra of H_2_O-DNA_hyd_, D_2_O-DNA_hyd_ (with and without drug, either cisplatin or Pd_2_Spm-8 µM) and DNA_lyoph_ were recorded at the Unidade de I&D Química-Física Molecular of the University of Coimbra (QFM-UC, Coimbra, Portugal), using a Bruker Optics Vertex 70 spectrometer (see details in the Supporting Information).

### 2.3. MicroRaman

The Raman microspectroscopy data of H_2_O-DNA_hyd_, D_2_O-DNA_hyd_ and DNA_lyoph_ (with and without drug) were acquired at QFM-UC (Portugal), in a WITec confocal Raman microscope system alpha300 R coupled to an Ultra-High-Throughput-Spectrometer UHTS 300 VIS-NIR, using the 532 nm line of a diode laser as the exciting radiation (see details in the Supporting Information).

### 2.4. INS

INS data were acquired at the ISIS Pulsed Neutron and Muon Source of the STFC Rutherford Appleton Laboratory (Didcot, United Kingdom), using the time-of-flight, high-resolution broad range spectrometer TOSCA [44,45,46] (see details in the Supporting Information). H_2_O-DNA_hyd_, D_2_O-DNA_hyd_ (with and without drug) and DNA_lyoph_ were measured. All data were taken at low temperature < 10 K.

The spectrum of the empty aluminium can was subtracted from the measured data for all DNA samples, with a view to better identify the low frequency vibrational features of the nucleic acid (which could be overlapped with the bands from the Al container).

### 2.5. QENS

The QENS experiments were performed on the High-Flux Backscattering spectrometer (HFBS) [47] (see details in the Supporting Information) at the NCNR (Gaithersburg, Maryland, MD, USA). Measurements were carried out for H_2_O-DNA_hyd_ and D_2_O-DNA_hyd_ (with and without drug) at low temperature (<10K) and at 298 K. A constant hydration degree was used (r.h. > 80%) to ensure that the measured variations in the QENS profiles were solely due to the effect of the drug and not to transitions induced by differences in the macromolecule’s hydration.

Fitting of the QENS spectra was performed with the program DAVE (version 2.6, developed at the National Institute of Standards and Technology (NIST) Center for Neutron Research (Gaithersburg, Maryland, MD, USA) [48] (see details in the Supporting Information).

## 3. Results and Discussion

**Scheme 1:**
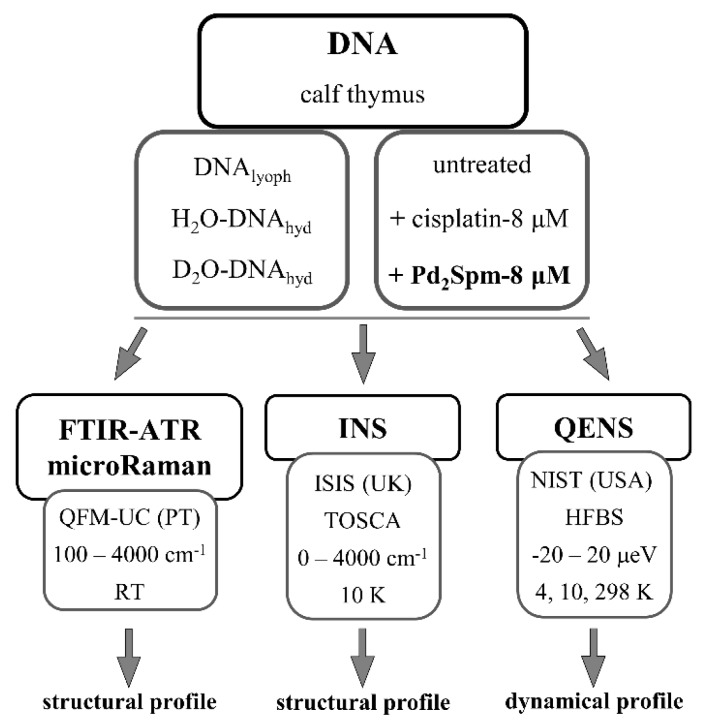
Schematic representation of the present study—samples under analysis and techniques used. (H_2_O-DNA_hyd_ and D_2_O-DNA_hyd_—DNA samples hydrated with H_2_O or D_2_O, respectively (r.h. > 80%); DNA_lyoph_: lyophilised DNA).

This study aims to achieve detailed information on the mode of action of the dinuclear Pd(II)-spermine anticancer agent Pd_2_Spm, in particular, regarding its effect on DNA’s conformational and dynamical preferences mediated by an impact on the nucleic acid’s hydration layer (leading to cytotoxicity). This comes as a continuation of previous studies of Pd_2_Spm-DNA interplay, using QENS coupled to synchrotron-based Fourier transform infrared spectroscopy (SR-FTIR) and extended X-ray absorption fine structure (SR-EXAFS) [12], that provided structural information as well as dynamical data at the picosecond timescale: from the drug-prompted changes in DNA’s conformation and the local environment of the absorbing Pd(II) centre in the drug-DNA adducts, to the drug-triggered variations in water mobility within DNA’s hydration shell. This complementary work probed the drug effect on DNA in a twofold way (Scheme 1): (i) assessing the drug impact on the nucleic acid’s slow dynamical processes (by QENS, in the nanosecond timeframe), such as motions from the phosphoribose backbone and the restricted hydration waters that were not observable in the picosecond timescale of the previous experiments; (ii) detecting (through Raman, FTIR and INS) DNA’s conformational changes, expected to be triggered by drug binding.

### 3.1. Drug Effect on DNA Conformation

**Figure 1:**
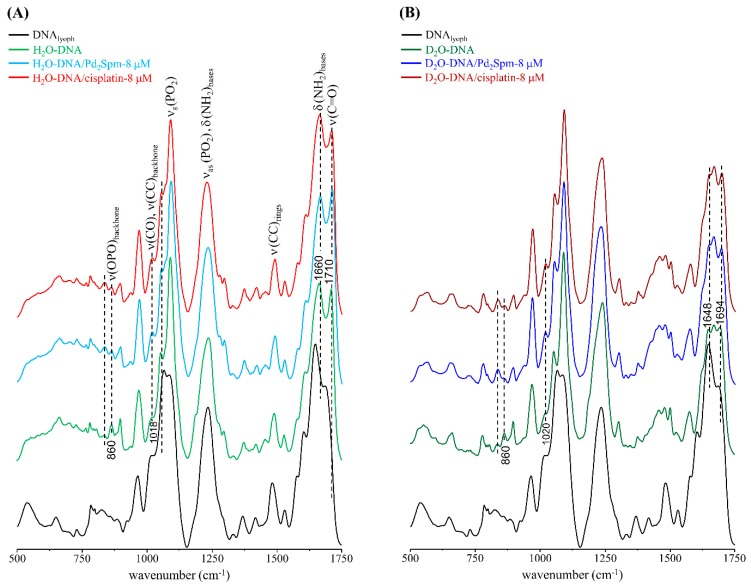
FTIR-ATR spectra (500–1750 cm^−1^) of DNA_lyoph_, H_2_O-DNA_hyd_ (**A**) and D_2_O-DNA_hyd_ (**B**), untreated and upon incubation (for 48 h) with cisplatin-8 µM or Pd_2_Spm-8 µM. (The main spectral changes are marked by dotted lines).

The infrared fingerprint region of the DNA samples (both H_2_O- and D_2_O-hydrated) clearly revealed drug-prompted spectral changes, namely regarding the bands from the nucleic acid’s phosphates (specifically ν(OPO), at ca. 860 cm^−1^), and from the amine and carbonyl groups (between 1600 and 1750 cm^−1^) (Figure 1). In particular, the 1710/1660 cm^−1^ peak ratio (ν(C=O)/δ(NH_2_)), which is known to increase with DNA’s hydration level [49], was found to be clearly affected by drug exposure mainly for the Pd_2_Spm-treated sample: varying from 0.966 for H_2_O-DNA to 0.918 and 1.036 for (H_2_O-DNA + cisplatin) and (H_2_O-DNA + Pd_2_Spm), respectively (Figure 1A). Comparison between the FTIR spectra of H_2_O-DNA_hyd_ and D_2_O-DNA_hyd_ (Figure 1A,B) shows major differences in the 1600 to 1750 cm^−1^ interval upon deuteration of the amine labile groups, as well as in the 1350 to 1500 cm^−1^ range that comprises the ν(CC) signals from the purine and pyrimidine rings.

The high wavenumber FTIR signature of lyophilised DNA, comprising the CH stretching bands (2800–2950 cm^−1^) as well as the signals from the ν(NH) and ν(OH) modes (*ca.* 3200–3420 cm^−1^), revealed a steady deviation of the ν(OH) band upon drug treatment: from 3400 cm^−1^ to 3406 and 3416 cm^−1^ for untreated and cisplatin- or Pd_2_Spm-exposed H_2_O-DNA_hyd_, respectively. This suggests a drug impact on the degree of hydrogen bonding within DNA’s hydration layer and between this and the biomolecule itself, which differs for each type of agent currently investigated—mononuclear Pt-compound (cisplatin) versus dinuclear Pd-complex (Pd_2_Spm). This disruption of the H-bonding network has been previously reported to have a significant effect on DNA’s vibrational profile [30].

**Figure 2:**
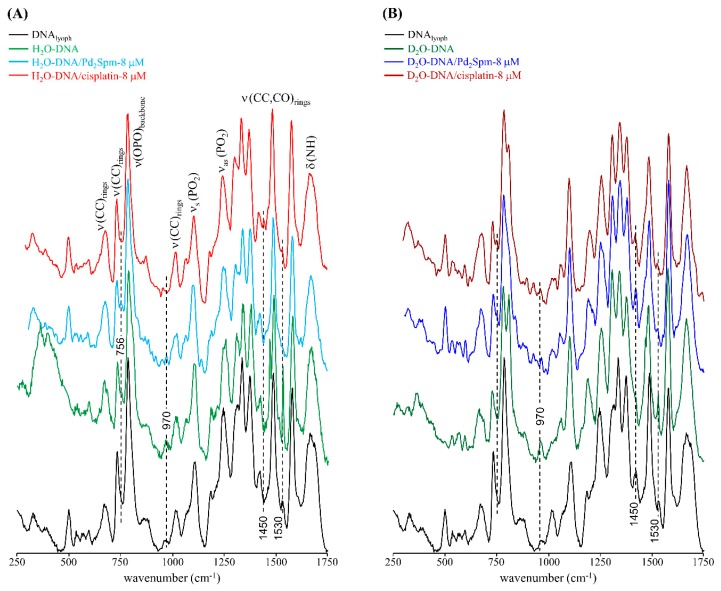
Raman spectra (250–1750 cm^−1^) of DNA_lyoph_, H_2_O-DNA_hyd_ (**A**) and D_2_O-DNA_hyd_ (**B**), untreated and upon incubation (for 48 h) with cisplatin-8 µM or Pd_2_Spm-8 µM. (The main spectral changes are marked by dotted lines.)

The drug-prompted effects on DNA’s conformation and hydration layer organisation were also reflected in the Raman spectra of drug-free versus drug-incubated DNA_hyd_, which showed variations mainly associated with the features ascribed to υ(OPO) (at 830 cm^−1^) and ν(CC)/(CO)_ring_ (namely, at approximately 756, 970, 1450 and 1530 cm^−1^), more noticeable for the D_2_O-hydrated samples (Figure 2).

These results are in accordance with previous microRaman and synchrotron-based FTIR studies on several human cancer cell lines as well as on DNA [11,12,13,15], that unveiled specific spectral biomarkers of the drug interplay with the nucleic acid (e.g., ν(OPO) and δ(NH_2_)) both through direct coordination to the double helix and via interference with the surrounding water molecules. The present data thus supports the covalent binding of these type of metal-based agents to DNA (predominantly at the N7 atoms from adenine and guanine) leading to a disruption of the double helix base pairing (which involves the corresponding NH_2_ groups), and to concomitant changes in the biomolecule’s backbone (reflected in the phosphate vibrational bands).

Drug-exposed DNA was probed by INS for the first time; this technique having been previously applied to hydrated DNA [30,32,50], but never for characterising the effect of a drug on the biopolymer. Following previous QENS experiments that revealed a clear effect of these anticancer compounds on the dynamical behaviour of DNA through an impact on its hydration layer, the present INS experiments aimed to monitor drug-elicited structural changes in the nucleic acid as well as within its hydration shell.

**Figure 3:**
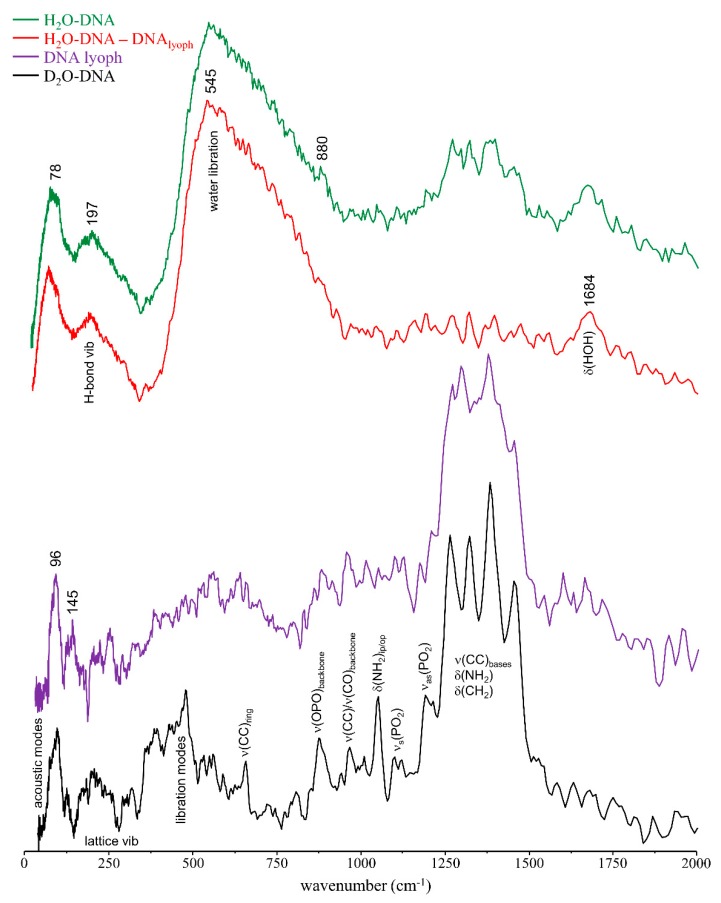
INS spectra (at 10 K) of DNA_lyoph_, D_2_O-DNA_hyd_, H_2_O-DNA_hyd_ and (H_2_O-DNA_hyd_–DNA_lyoph_).

The INS spectra of H_2_O-DNA_hyd_ is represented in Figure 3: the low wavenumber, acoustic region (below 120 cm^−1^) comprises the lattice modes; the translational area (160–330 cm^−1^) contains the ring puckering and intermolecular H-bond vibrations (water-water and water-DNA); and the very intense water librational band lies between 400 and 950 cm^−1^, with a maximum at ~550 cm^−1^, in good accordance with the results previously obtained by the authors for intracellular water in human breast cancer cells [15]. The lowest energy signal was reported to be associated to DNA’s hydration level [30,34], the currently observed band at ~78 cm^−1^ being consistent with a highly hydrated sample (>80%). The region between 1250 and 1500 cm^−1^ comprises a clear set of bands mainly due to CH_2_ deformations and ring stretching modes from the purine and pyrimidine bases, which have not been reported before (and could be presently detected thanks to the extremely high sensitivity of the recently upgraded TOSCA spectrometer [44,45,51]. In addition, a signal was detected at 880 cm^−1^ ascribed to υ(CC) from the ribose rings, and the δ(H_2_O)_sciss_ mode was distinctly observed at 1684 cm^−1^. The INS profile of H_2_O-DNA_hyd_ upon subtraction of lyophilised DNA is also depicted in Figure 3, allowing to better discriminate the features from DNA’s hydration water, i.e., the bands from water molecules directly interacting with the nucleic acid’s double helix: acoustic modes at ca. 78 cm^−1^, water–water hydrogen bond vibrations at 197 cm^−1^, and the broad and intense water librational signal peaking at 545 cm^−1^ (that does not display the left-edge characteristic of ice). In addition, the deformation band from water is clearly detected at l684 cm^−1^. In turn, the vibrational profile of D_2_O-DNA_hyd_ (with a deuterated hydration layer) clearly reveals the characteristic bands of the nucleic acid molecule, since the intense water librational mode is absent (Figure 3) and displays an expected resemblance to the vibrational signature of the biomass depicted for DNA_lyoph_.

**Figure 4:**
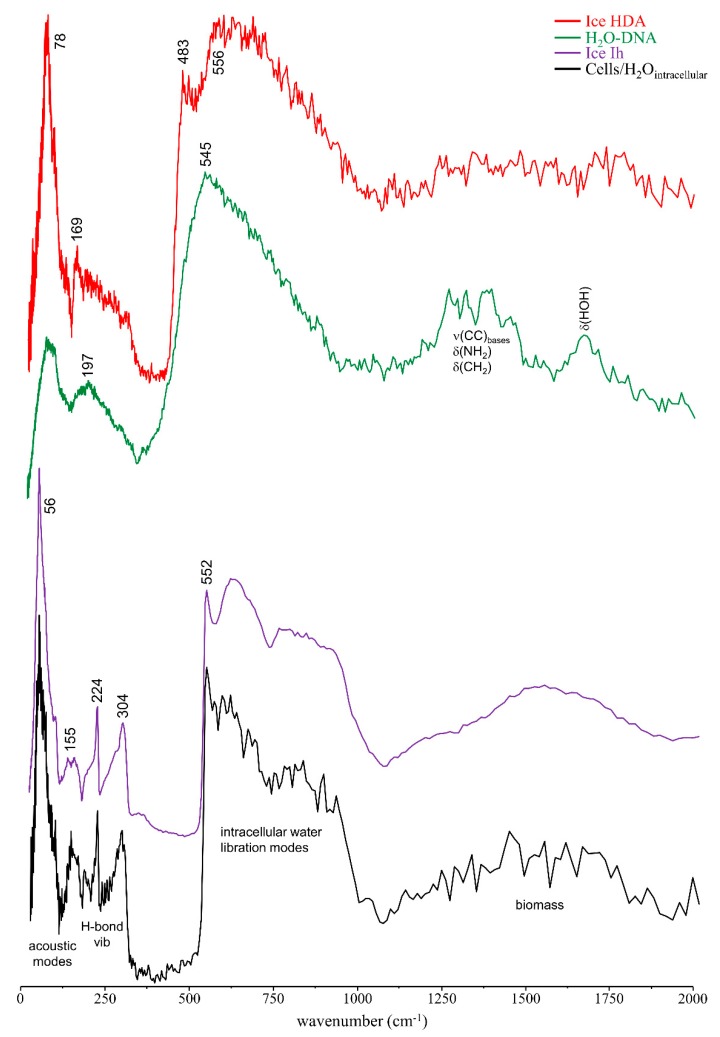
INS spectra (at 10 K) of H_2_O-DNA_hyd_ and human breast cancer cells (MDA-MB-231) [15]. The INS data for hexagonal ice (Ih) and high density amorphous ice (HDA) [52,53] are also shown for comparison.

Interestingly, while the water librational band formerly detected for intracellular water in human cancer cells [15] was identical to the H-bonded tetrahedral network of ice Ih, the librational feature presently observed for H_2_O-DNA_hyd_ is similar to that detected for high density amorphous (HDA) ice (Figure 4), which differs significantly from ordinary ice Ih: the two distinct peaks at 224 and 304 cm^−1^ characteristic of the H-bonded network of water in hexagonal ice are absent, and the librational lower energy edge is red-shifted relative to ice Ih (483 cm^−1^ as compared to 552 cm^−1^, respectively) [52,53]. This clearly evidences the different organisation of water molecules within a biomolecule’s hydration layer (forming a quite rigid H-bonded network) when compared with the water arrangement in the cellular cytoplasm (intracellular medium), which is more similar to bulk water. In the hydration shell, water molecules in contact with the nucleic acid are subject to stronger interactions, such as electrostatic close-contacts with the hydrophilic groups at the surface of DNA’s double helix.

**Figure 5:**
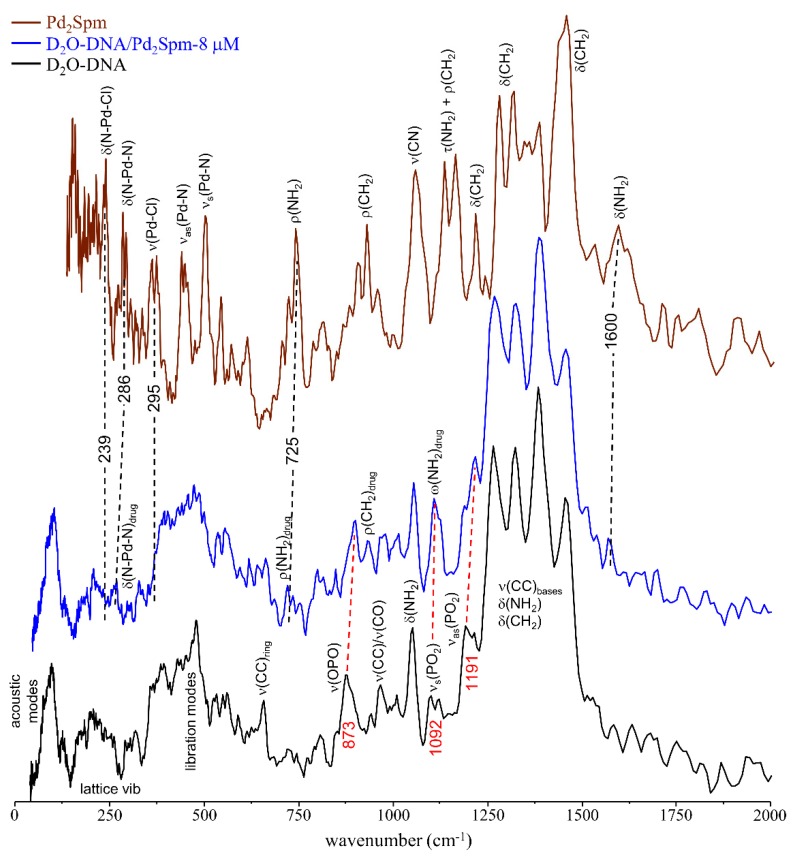
INS spectra (at 10 K) of D_2_O-DNA_hyd_ untreated and upon incubation (for 48 h) with Pd_2_Spm-8 µM. The spectrum of the free drug is also shown [17]. (The main drug-triggered vibrational changes are shown by red and black dashed lines and wavenumbers for DNA’s and Pd_2_Spm vibrational modes, respectively).

Upon incubation with the drugs (Pd_2_Spm and cisplatin at 8 µM, for 48 h), clear changes were detected in the INS profile of D_2_O-DNA_hyd_ (Figure 5 and Figure S1/Supplementary Material), revealing both the drug effect on the nucleic acid and variations in the drug molecule prompted by interaction with DNA, which takes place primarily via covalent binding to the nitrogen atoms of the purine and pyrimidine bases. For Pd_2_Spm, in particular, the spectral profile of the drug-exposed DNA reveals a few characteristic bands from the Pd-complex, some of them shifted relative to the free complex due to drug-binding to the double helix, namely δ(N-Pd-N) and ρ(NH_2_) which were observed at 264 and 716/730/749 cm^−1^ as compared to 286 and 707/725/745 cm^−1^ in unbound Pd_2_Spm [17], and δ(NH_2_) that was detected at 1570 cm^−1^ in Pd_2_Spm–D_2_O-DNA_hyd_ as opposed to 1600 cm^−1^ in free Pd_2_Spm. Additionally, the modes involving the metal-to-chloride bonds within the drug, namely δ(N-Pd-Cl) and ν(Pd-Cl) at 239 and 295/361 cm^−1^, respectively, were found to disappear upon interaction with DNA, as expected upon intracellular Cl hydrolysis prior to DNA metallation. Variations were also evidenced in DNA’s typical features, particularly those assigned to the phosphate groups: ν(OPO) shifted from 873 to 897 cm^−1^ upon drug exposure, ν_as_(PO_2_) varied from 1191 to 1216 cm^−1^ and ν_s_(PO_2_) (at 1092 cm^−1^) was overruled by the drug’s ω(NH_2_) band. The nucleic acid’s lattice modes and intrahelical vibrations (DNA breathing), detected below 500 cm^−1^, also revealed changes upon drug treatment as expected in the light of the known drug-elicited conformational rearrangement of the biomolecule: severe damage of the native B-conformation, through disruption of the double helix H-bonded base-pairs (DNA unzipping) and of the hydrophobic interactions between base rings (purines and pyrimidines). Actually, the low frequency DNA modes, which are critical to biological function and cooperative in nature, are very sensitive to structural reorientations, and therefore particularly prone to be affected by drug interaction. On the other hand, the vibrational signature within the 1220–1500 cm^−1^ interval (ν(CC), δ(NH_2_) and δ(CH_2_) DNA modes) was not significantly disturbed by drug-binding.

### 3.2. Drug Effect on DNA Dynamics

Apart from the conventional interaction of metal-based agents with DNA through covalent binding (leading to conformational rearrangements), these types of drugs have been previously shown to cause a significant reorganisation of the water molecules surrounding the biomolecule, prompting an increased mobility within its hydration layer [12,15,16,19]. Upon these drug-elicited perturbations, DNA is rendered less-functional or fully nonfunctional, which leads to cell growth inhibition and cell death. The dynamical behaviour of water is, therefore, a sensitive and reliable probe of changes induced by an external agent. It was formerly shown that deuteration of DNA’s hydration shell or the absence of a hydration layer (in lyophilised DNA samples) hinder the drug impact on the macromolecule [12], evidencing the key role of mobile hydrogens and hydration water molecules as key mediators in drug interaction with the biopolymer leading to cytotoxicity. Actually, hydration shell dynamics and conformational reorganisation of the biomolecule have been reported to be intertwined, the characteristic DNA dynamic transition (at 222 ± 2 K [54]) being driven by mobility changes within its hydration shell [38,54,55]. Furthermore, the dynamical processes within DNA’s hydration shell, containing a significant population of rapidly diffusing water molecules, are modified by the boundary conditions set by the biomolecule. It spans a broad range of timescales (from the ns to the ps) due to the high heterogeneity of DNA’s topography and chemical nature of its exposed sites, and to the diversity of water-biopolymer interactions (e.g., electrostatics, hydrophobic and H-bonding) [56,57].

In hydrated DNA there are several dynamical components to consider in the pico-nanosecond timescales: (i) reorientation of water molecules in the primary hydration layer (fast translations according to a non-diffusive jump reorientation model [55,58,59]); (ii) fast localised motions, namely rotations ascribed to CH_3_ and NH_2_ groups of the molecule (not involved in hydrogen-bonds); (iii) diffusive-like motions of the hydration water molecules confined within DNA’s “spine of hydration”—highly ordered water molecules that penetrate deeply into the minor groove of B-DNA duplexes, mainly in the narrow AT-rich region, with a relatively long residence time [29,60,61,62,63] (the analogous to the internal hydration pockets of globular proteins); and (iv) global relaxation of the nucleic acid’s backbone—slow cooperative motions of the base-pairs and phosphoribose segments. Whereas components (i) and (ii) occur in the picosecond timescale and have been previously studied [12], (iii) and (iv) are slower motions that take place mostly in the nanosecond timescale. These will be discussed below.

**Figure 6:**
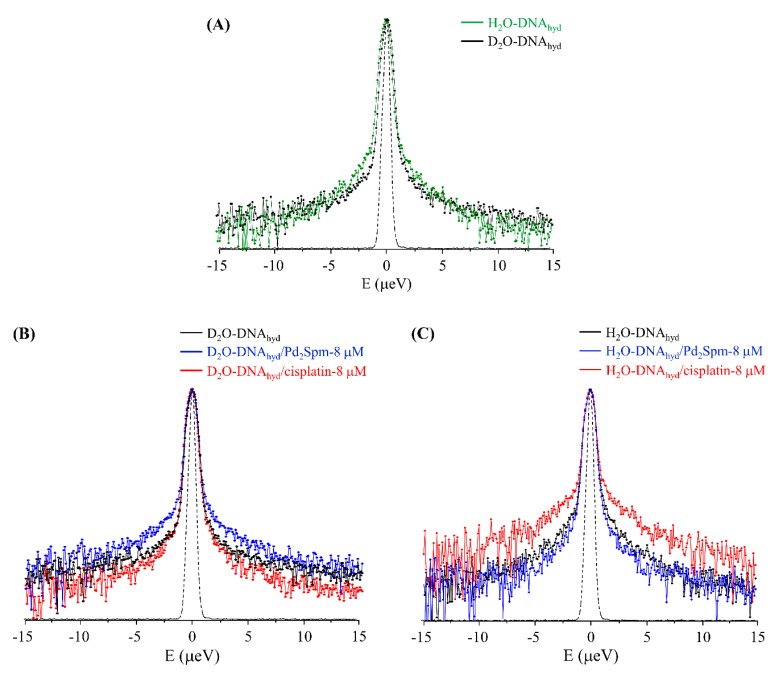
QENS profiles (298 K, averaged over the entire Q range, logarithmic scale) for: (**A**) H_2_O-DNA_hyd_ *vs* D_2_O-DNA_hyd_; (**B**,**C**) untreated and drug-exposed (8 µM) D_2_O-DNA_hyd_ and H_2_O-DNA_hyd_, respectively.The spectra were normalised to maximum peak intensity. The black dashed line represents the instrument resolution.

Although the incoherent scattering signal measured for the H_2_O-DNA_hyd_ samples is due to both the water hydrogens from the spine of hydration and to the molecule’s backbone (non-labile hydrogens), the QENS spectrum obtained for D_2_O-DNA_hyd_ reflects only the latter (all the waters in the external hydration sheath being deuterated). Thus, as expected, D_2_O-DNA_hyd_ displays slower dynamics than H_2_O-DNA_hyd_ (Figure 6A), the difference in the QENS profiles being ascribed to the motions from the water molecules within DNA’s minor groove. Therefore, the drug’s effect on the nucleic acid’s inner hydration (constrained water molecules within the minor groove) was probed in H_2_O-DNA_hyd_ while the impact on the backbone dynamics was monitored in D_2_O-DNA_hyd_.

**Figure 7:**
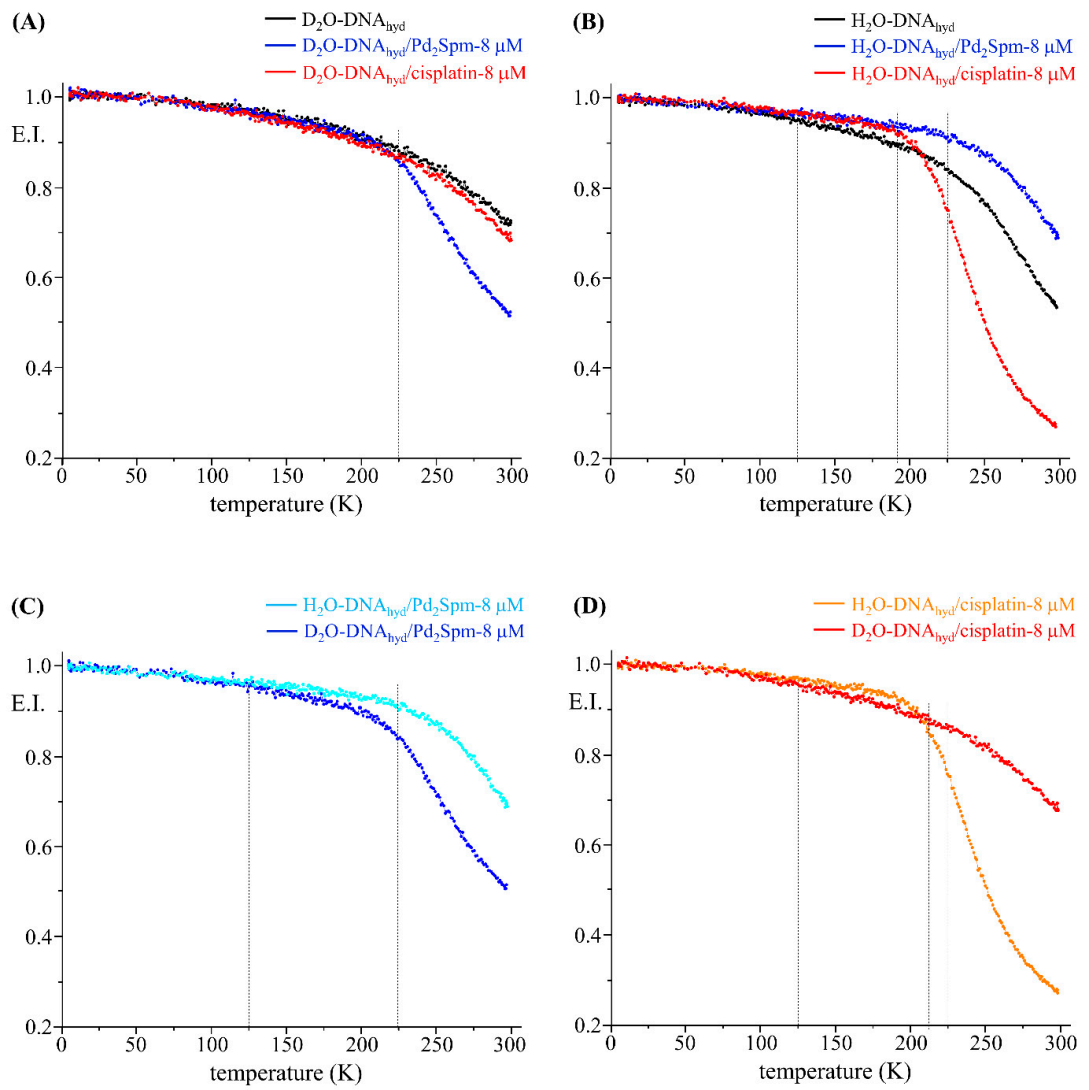
Elastic scan plots (5–298 K) for untreated and drug-exposed (8 µM) D_2_O- and H_2_O-DNA_hyd_.

The QENS profiles of drug-free and drug-exposed D_2_O- and H_2_O-DNA_hyd_ are represented in Figure 6B,C, whereas Figure 7A–D comprises the corresponding elastic scan plots. In elastic scan mode, only the elastically scattered neutrons are recorded and any drop in intensity with temperature indicates an onset of dynamical modes that can be resolved by the instrument. These results evidence clear distinct effects of the two tested drugs (cisplatin and Pd_2_Spm) on both DNA and water, at the nanosecond timescale. Regarding the impact on DNA’s backbone, the Pd-agent has a much larger effect (D_2_O-DNA_hyd_, Figure 6B and Figure 7A,C) prompting a significantly increased mobility, as compared with cisplatin that has a very weak impact (as also evidenced from the mean square displacements, determined from the elastic fixed window scans of the incoherent neutron scattering, Figure S2/Supplementary Material). This is hypothesised to be due to the predominant effect of the Pd_2_Spm on DNA’s backbone—these types of dinuclear complexes are capable of establishing two covalent bonds between their metal centres and DNA bases [11], whereas cisplatin and similar mononuclear agents can only interact with the nucleic acid via one covalent bond per drug molecule. Thus, the distinct mode of action of the mononuclear (Pt) versus dinuclear (Pd) drugs currently probed is clearly reflected in these experimental results: while cisplatin binds preferentially to the N_7_ atoms of adenine and guanine, yielding short-range intrastrand adducts [64], Pd_2_Spm’s interaction with DNA (at the same coordination sites) gives rise to dinuclear long-range intra- and interstrand interactions, therefore prompting a greater conformational disruption in the nucleic acid (in agreement with the higher cytotoxicity formerly measured for this compound against several types of human cancer cells, as compared to cisplatin [8,9]).

Figure 7C,D also indicates that the secondary macromolecular relaxations of DNA, not very closely linked to water, activate quite early (at ~125 K) and are not affected by exposure to the drugs. The main dynamical transition (as referred to in the literature, highly correlated to the water dynamics and the fragile-to-strong crossover at 222 ± 2 K [28,54,65]) is also not strongly influenced by the drugs, however the temperature at which it occurs changes slightly. In the untreated samples this transition takes place at ~222 K and is similar in the Pd_2_Spm-treated samples. However, cisplatin lowers the transition temperature to ~200 K (Figure 7B). These observations agree with the stronger impact of the Pt-drug on DNA’s hydration shell (as compared to Pd_2_Spm), and corroborate the recognised influence of the hydration layer on a biomolecule’s dynamical profile—variations in the mobility of the hydration water driving the dynamic transition of the biopolymer [38,54,55].

We now discuss the influence of these drugs on the water dynamics. The response is in fact opposite to that for DNA, Pt-based cisplatin showing a much more significant effect on the hydration water dynamics than Pd_2_Spm (H_2_O-DNA_hyd_, Figure 6C and Figure 7B,D). These results suggest that cisplatin acts on the nucleic acid mainly via its hydration waters (in the minor groove) while Pd_2_Spm has an effect mostly mediated by dynamical changes in the biomolecule’s backbone as explained previously. The current results, at the nanosecond timescale, are in accordance with former measurements performed by the authors on the dynamics of water at the picosecond time window that revealed a stronger impact of cisplatin on DNA’s hydration layer relative to Pd_2_Spm [12]. Therefore, cisplatin has a strong disruptive effect on the organised water shell (inducing a higher flexibility as compared to drug-free DNA).

Furthermore, at temperatures below 200 K, the data for H_2_O-DNA_hyd_ with both drugs (elastic scan plots in Figure 7B and corresponding mean square displacements in Figure S2/Supplementary Material) reflect a dynamical behaviour that is even slower than the one corresponding to drug-free DNA. This suggests that the drugs inhibit the mobility of the supercooled/confined water, probably the hydration water molecules that are more strongly associated with the biomolecule.

The experimental QENS data was fit using one δ-function (elastic component) convoluted with the line shape of the instrument and Lorentzian functions to represent the quasielastic contributions (as well as a sloping background): while D_2_O-DNA was fitted with one narrow Lorentzian (Γ_global_) ascribed to the slow hydrogen motions of the biomolecule’s backbone, a second, broader, Lorentzian (Γ_local_) was added in H_2_O-DNA to represent the faster dynamics of the water molecules within DNA’s spine of hydration (Figure S3/Supplementary Material). The very slow global motions of the macromolecule were defined by a Delta function (slower than the longest observable time determined by the instrument resolution).

**Figure 8:**
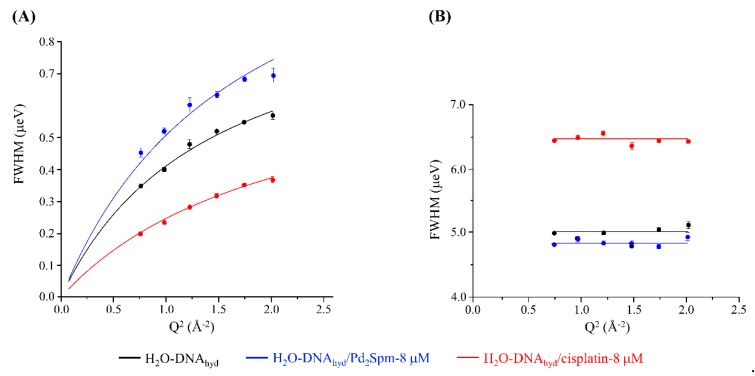
Variation of the full widths at half-maximum (FWHM) with Q^2^ for untreated, and Pd_2_Spm- and cisplatin-treated H_2_O-DNA_hyd_ (at 8 µM and 298 K): Lorentzian functions representing the slow backbone dynamics (Q-dependent) (**A**) and the water motions within the spine of hydration (Q-independent) (**B**). Error bars throughout the text represent one standard deviation unless defined otherwise.

The Q-dependence of the full width at half-maximum (FWHM = Γ) of the Lorentzian functions representing the dynamical components from DNA’s backbone and water molecules constrained within the minor groove are represented in Figure 8. Although the former displays a Q-dependent behaviour, being described appropriately by a translational jump reorientation model (according to Equation (7), Supplementary Material), the spine of hydration waters exhibit a Q-independent diffusive dynamics in accordance with their restricted mobility (i.e., they are localised in space) [66]. Actually, while for unconstrained hydration water molecules, binding almost exclusively to the outer surface of the double helix, a non-diffusive jump reorientation model accurately describes the water translational dynamics as previously shown [12]; this model fails to represent the motions of the H_2_O molecules confined within DNA’s spine of hydration. In a biological matrix, these water molecules can exchange with the external medium (cellular cytoplasm) and the kinetics of this water exchange should influence the binding of drugs to the nucleic acid.

Table 1 comprises the values of the diffusion coefficients (D_T_) and residence times (τ_T_) for the slow dynamics of DNA’s backbone and the motions of the water molecules within DNA’s spine of hydration, for the two drugs tested (at 8 µM and 298 K). These results quantitatively reflect the effect of the metal-based agents on DNA’s dynamical behaviour in the nanosecond timeframe. The flexibility of the molecule’s backbone is significantly increased by Pd_2_Spm binding, as opposed to cisplatin—D_T_ and τ_T_ values equal to 1.0621 ± 0.0291 × 10^−5^ cm^2^ s^−1^/0.6607 ± 0.0221 ns, 1.2267 ± 0.0962 × 10^−5^ cm^2^ s^−1^/0.4796 ± 0.0594 ns and 0.5532 ± 0.03217 × 10^−5^ cm^2^ s^−1^/0.8557 ± 0.0784 ns, respectively, for drug-free, Pd_2_Spm-D_2_O-DNA_hyd_ and cisplatin-D_2_O-DNA_hyd_. An opposite trend was found for the faster water motions within DNA’s spine of hydration, which are rendered faster by cisplatin while they are only slightly less mobile in the presence of Pd_2_Spm: τ_T_ equal to 0.1995 ± 0.0223, 0.2067 ± 0.0112 and 0.1550 ± 0.0173 ns for drug-free, Pd_2_Spm- and cisplatin-exposed H_2_O-DNA_hyd_, respectively. This is in accordance with the distinct predominant effect of each drug either on DNA’s backbone or on its minor groove hydration, as reflected in the respective QENS profiles (Figure 7A,B). Interestingly, the significant Pd_2_Spm-prompted perturbations of the biomolecule’s backbone were found to have a small effect on the water dynamics within the confined hydration pockets (minor groove).

The results from this work are in line with previous nuclear magnetic relaxation dispersion studies on hydrated DNA, that report residence times shorter than 1 ns for the water molecules within the minor groove spine of hydration—approximately 0.2 ns at 27 °C and ca. 0.6 ns at 10 °C [62]. These values are much longer than those from DNA’s external hydration shell—10 ps [12,32] and between 7.4 and 8.4 ps for drug-exposed DNA [12], which, in turn, are one order of magnitude higher than the correlation time for bulk water (1.25 ps [67]) reflecting the greatly reduced mobility of water molecules adsorbed to DNA’s surface.

## 4. Conclusions

The impact of a new metal-based anticancer agent (Pd_2_Spm) on DNA was studied, in both H_2_O- and D_2_O-hydrated samples, and compared with the clinically used Pt-drug cisplatin. Conventional and unconventional drug-target interactions were probed, respectively, through covalent binding and via interference with the hydration waters. The combined use of QENS and complementary vibrational spectroscopy techniques (FTIR-ATR, microRaman and INS) allowed us to obtain detailed data on the drug-triggered changes in DNA at the conformational and dynamic levels.

Drug-induced spectral changes were clearly detected by both FTIR and Raman (in H_2_O- and D_2_O-DNA_hyd_), mainly in the features assigned to the phosphates, and the amine and carbonyl groups from the nitrogenous bases. In addition, the FTIR data revealed a different drug impact on the degree of hydrogen bonding within DNA’s hydration layer, as well as between this and the biomolecule itself. The INS data obtained for untreated and drug treated-DNA allowed to unveil changes in both the drug and the target: regarding the former, the vibrational modes associated to the metal-to-chloride bonds were found to disappear upon DNA binding; for the latter, the most significant variations were seen in the bands from the phosphate group and the low frequency DNA vibrations (lattice and breathing modes).

In the light of previous studies by the authors that showed a drug impact on the picosecond dynamics of the water molecules at the surface of hydrated DNA, the current QENS measurements in the nanosecond timescale probed the slower motions of the nucleic acid’s backbone and of the water molecules constrained within its “spine of hydration”, expected to be affected by these DNA-targeting compounds. Clearly distinct effects were observed for cisplatin and Pd2Spm, the former having a predominant impact on DNA’s spine of hydration while the latter had a higher influence on the backbone dynamics.

This innovative assessment of the impact of a chemotherapeutic agent on the dynamics of vital biomolecules (such as DNA) through an effect on their hydration layers, as well as on their native structure, is key for a thorough understanding of the particular mechanism of action of dinuclear metal agents such as Pd_2_Spm. This will improve the understanding of the molecular basis of cytotoxicity of these kinds of antitumour drugs, thus contributing to the design of chemotherapeutic agents with optimised efficacy, that may act on more than one target: (i) directly binding to DNA, causing disruption of its native conformation and prompting biofunctional disability; (ii) interacting with its hydration waters (both at the surface and within inner pockets) eliciting changes in the biopolymer’s dynamical profile. Thus, apart from the generally accepted DNA drug-binding sites (purine and pyrimidine bases), water in the biopolymer’s hydration layers may constitute an important and non-negligible therapeutic target. Both effects should be responsible for a disruption of DNA’s functional conformation, thus triggering cell growth inhibition and death.

## Figures and Tables

**Table 1:** Translational diffusion coefficients (D_T_) and relaxation times (τ_T_) of water for untreated and Pd_2_Spm- and cisplatin-treated (at 8 µM) D_2_O-DNA_hyd_ and H_2_O-DNA_hyd_ (at 298 K) for the dynamics of DNA’s backbone (Q-dependent) and the water motions within the spine of hydration (Q-independent). The translational diffusion coefficients and relaxation times of hydration water and fast localised motions in the picosecond timescale, formerly obtained for these systems, are also shown (shaded columns).

Sample	Backbone		H_2_O/Spine Hydration	H_2_O/Hydration Shell *^a^*		Fast Localised Motions *^a^*
	D_T_ (×10^−5^ cm^2^ s^−1^)	τ_T_ (ns)	τ_T_ (ns)	D_T_ (×10^−5^ cm^2^ s^−1^)	τ_T_ (ps)	τ_T_ (ps)
D_2_O-DNA_hyd_	1.0621 ± 0.0291	0.6607 ± 0.0221	_	_	_	_
D_2_O-DNA_hyd_ + cisplatin	0.5532 ± 0.0322	0.8557 ± 0.0784	_	_	_	_
D_2_O-DNA_hyd_ + Pd_2_Spm	1.2267 ± 0.0962	0.4796 ± 0.0594	_	_	_	_
H_2_O-DNA_hyd_	1.0621 ± 0.0291	0.6607 ± 0.0221	0.1995 ± 0.0223	0.723 ± 0.002	10.13 ± 0.32	3.591 ± 0.006
H_2_O-DNA_hyd_ + cisplatin	0.5532 ± 0.0322	0.8557 ± 0.0784	0.1550 ± 0.0173	0.901 ± 0.004	7.40 ± 0.36	3.270 ± 0.003
H_2_O-DNA_hyd_ + Pd_2_Spm	1.2267 ± 0.0962	0.4796 ± 0.0594	0.2067 ± 0.0112	0.744 ± 0.004	8.38 ± 0.86	3.387 ± 0.002

*^a^* From ref. [12].

## Supplementary Materials

The following are available online at, Figure S1: INS spectra (at 10 K) of D_2_O-DNA_hyd_ untreated and upon incubation (for 48 h) with cisplatin-8 µM. (The main drug-triggered vibrational changes in DNA are shown by dashed lines). Figure S2: Temperature variation of the mean-squared displacements for untreated and drug-exposed (8 µM) D_2_O- and H_2_O-DNA_hyd_. Figure S3: QENS spectra (298 K) for H_2_O-DNA_hyd_—untreated (A) and Pd_2_Spm-treated (8 μM) (B)—fitted using two Lorentzians and one Delta functions, at some typical Q values.
