# Supplementary material for: A New Look into the Mode of Action of Metal-Based Anticancer Drugs
Source: Molecules. 2020 Jan 7;25(2):246. doi: 10.3390/molecules25020246 (PMC7024343; doi:10.3390/molecules25020246)
Supplement: Supplementary file 1 [file molecules-25-00246-s001.pdf]

Supplementary Material

A New Look into the Mode of Action of Metal-based Anticancer Drugs

M. Paula M. Marques [1,2], Ana L.M. Batista de Carvalho [1,*], Adriana P. Mamede [1], Asha Dopplapudi [3], Svemir Rudić [3], Madhusudan Tyagi [4], Victoria Garcia Sakai [3] and Luís A.E. Batista de Carvalho [1]

1 Unidade de I&D Química-Física Molecular, Department of Chemistry, University of Coimbra, Coimbra 3004-535, Portugal
2 Department of Life Sciences, University of Coimbra, Coimbra 3000-456, Portugal
3 ISIS Facility, STFC Rutherford Appleton Laboratory, Chilton, Didcot OX11 0QX, UK
4 NIST Center for Neutron Research, Gaithersburg, Maryland 20899 and University of Maryland, College Park, Maryland 20742, USA

Table of Contents

Materials and Methods

Chemicals
Synthesis and Characterization of Pd_2Spm
INS Fundamentals and Measurements
QENS Fundamentals and Measurements
FTIR-ATR Measurements
MicroRaman Measurements
Data Analysis

Figures

Figure S1 – INS spectra (at 10 K) of D_2O-DNA$_{hyd}$ untreated and upon incubation (for 48 h) with cisplatin-8 µM.
(The main drug-triggered vibrational changes in DNA are shown by dashed lines).

Figure S2 – Temperature variation of the mean-squared displacements for untreated and drug-exposed (8 µM) D_2O- and H_2O-DNA$_{hyd}$.

Figure S3 – QENS spectra (298 K) for H_2O-DNA$_{hyd}$ – untreated (A) and Pd_2Spm-treated (8 µM) (B) – fitted using two Lorentzians and one Delta functions, at some typical Q values.

References

Materials and Methods

Chemicals

Deoxyribonucleic acid (sodium salt from calf thymus, type I, fibers), N,N´-*bis*(3-aminopropyl)-1,4-diaminobutane (spermine, free base), *cis*-dichlorodiammine platinum(II) (cisplatin, >98%), phosphate buffered saline (PBS), potassium tetrachloropalladate(II) (K_2PdCl_4, >99.9%), as well as solvents, inorganic salts and acids (of analytical grade) were purchased from Sigma-Aldrich Chemical S.A. (Sintra, Portugal).

Synthesis and Characterisation of Pd_2Spm

The Pd_2Spm complex was synthesised according to published procedures [1] optimised by the authors [2]: 2 mmol of K_2PdCl_4 were dissolved in a minimal amount of water and an aqueous solution containing 1 mmol of spermine was added dropwise under continuous stirring. After 24 h, the resulting orange powder was filtered off and washed with acetone, needle-shaped crystals having been obtained by recrystallization from water (yield 68%). The newly synthesised compound was fully characterised (and tested as to its purity) by elemental analysis and vibrational spectroscopy – FTIR, Raman and inelastic neutron scattering (INS), which were compared with the previously calculated vibrational profiles (by *ab initio* methods [2]). Elemental analysis, Found (for $Pd_2(C_{10}N_4H_{26})Cl_4$): C: 21.2%; H: 4.7%; N: 9.6%, Cl: 25.9%; Calculated – C: 21.5%; H: 4.6%; N: 9.9%, Cl: 25.6%.

INS Fundamentals and Measurements

Inelastic neutron scattering (INS) is an extremely useful technique for probing hydrogenous materials, the intensity of each vibrational transition being expressed, for a given atom, by the dynamic structure factor

$$\mathbf{S_i^*(Q, \nu_k)} = \frac{(\mathbf{Q^2 u_i^2})\sigma}{\mathbf{3}} \mathbf{exp}\left(-\frac{\mathbf{Q^2 \alpha_i^2}}{\mathbf{3}}\right) \qquad (1)$$

where Q ($Å^{-1}$) is the momentum transferred to the sample, ν_k is the energy of a vibrational mode, u_i(Å) is the displacement vector of atom i in mode k, σ is the neutron scattering cross section of the atom, and α_i(Å) is related to a mass-weighted sum of the displacements of the atom in all vibrational modes. There are no selection rules for INS (in contrast to FTIR and Raman) which yields all the fundamental modes, overtones and combination bands for the analyzed samples.

The INS measurements were performed at the ISIS Pulsed Neutron and Muon Source of the STFC Rutherford Appleton Laboratory (United Kingdom), using the time-of-flight, high resolution broad range spectrometer TOSCA [3-6].

H_2O-DNA_{hyd} and D_2O-DNA_{hyd} samples were measured, with and without drug (cisplatin and Pd_2Spm at 8 μM), as well as commercial (lyophilised) DNA (DNA_{lyoph}). These (1-2 g) were wrapped in aluminium foil and fixed onto 4×4 cm thin walled aluminium cans. To reduce the impact of the Debye-Waller factor (the exponential term in equation (1)) on the observed spectral intensity, the samples were cooled to 5-10 K. Data were recorded in the energy range 0 to 4000 cm^{-1}.

QENS Fundamentals and Measurements

Quasi-elastic incoherent neutron scattering (QENS) typically analyses the incoherent scattering signal resulting from a variety of atomic motions ranging from fast vibrational and rotational localised modes, to slower diffusional modes, motions taking place at pico- to nanosecond timescales, and exploits the high sensitivity of neutrons to hydrogen (with a much larger incoherent scattering cross section compared to that of other elements). The QENS signal, the so-called dynamic structure factor,

S(Q,ω), is due to a variety of dynamical processes (which fall in the spectrometer's time window), from fast localised modes including vibrations and rotations to slower global translational motions. It arises from energy (ħω) and momentum (Q) exchanges between the neutrons and the atoms within a given spectrometer resolution, and is detected as a broadening about an elastic line of energy exchange ≈0. The measured signal can be mathematically expressed as:

$$S_{measured}(Q,\omega)=\exp\left(-\frac{\hbar\omega}{2k}\right)R(Q,\omega)\otimes S(Q,\omega) \quad (2)$$

where exp[-ħω/2kT)] is a detailed balance parameter, and R(Q,ω) is the instrument´s resolution function (experimentally obtained) which is convoluted with the scattering model (S(Q,ω)) that describes the dynamical behaviour of the sample. In hydrogenous biological samples, S(Q,ω) is dominated by the incoherent scattering of the hydrogen atoms (much larger than the coherent or incoherent scattering cross section of any other atom) and are approximated as the convolution of vibrational, rotational and translational components (assumed as independent motions),

$$S_{inc}(Q,\omega)=S_{vib}(Q,\omega)\otimes S_{rot}(Q,\omega)\otimes S_{trans}(Q,\omega) \quad (3)$$

Strictly in the elastic and quasielastic regions,

$$S_{inc}(Q,\omega)=\exp(-Q^2\langle u^2\rangle)\left[A_0(Q)\delta(\omega)+\left(1-A_0(Q)\right)L(Q,\omega)\right] \quad (4)$$

where the exponential term is the Debye-Waller factor, $A_0(Q)\delta(\omega)$ is the elastic contribution due to motions slower than the longest observable time as defined by the energy resolution of the spectrometer, and the second term in the equation corresponds to the quasielastic component. Sinc(Q,ω) provides time/space information on the system probed, on the timescale of the dynamical processes (through the neutron energy transfer, ω), and on the spatial extent of these processes (from the momentum scattering transfer, Q). Once the QENS data in the time-domain is represented by an exponential, it can be approximated in the energy domain by Lorentzian functions of different widths,

$$L(x)=\frac{1}{\pi}\frac{\Gamma}{\Gamma^2+\omega^2} \quad (5)$$

Γ being the full width at half-maximum (FWHM=2xHWHM (half-width at half-maximum)). These Lorentzian functions describe motions occurring on different timescales, detailed information on each dynamic component being retrieved from the Q-dependency of Γ.

The QENS measurements were performed at the National Institute of Standards and Technology (NIST) Centre for Neutron Research (Gaithersburg, Maryland, USA), on the High-Flux Backscattering spectrometer (HFBS) [7] which affords a 0.8 μeV energy resolution (FWHM) and with the Doppler drive set for an energy transfer window of +/- 15μeV. Experiments were carried out at temperatures of < 10 K and 298 K. Elastic scans were measured at a cooling rate of 1 K/min starting from 5 K.

The samples – H_2O-DNA_{hyd} and D_2O-DNA_{hyd}, with and without drug – were mounted in indium-sealed Al flat cans. A vanadium sample (purely incoherent elastic scatterer) was also measured to correct for detector efficiency.

FTIR-ATR Measurements

The FTIR-ATR spectra were acquired at the "Molecular Physical-Chemistry" R&D Centre of the University of Coimbra (QFM-UC, Coimbra, Portugal), using a Bruker Optics Vertex 70 FTIR spectrometer purged by CO_2-free dry air and a Bruker Platinum ATR single reflection diamond accessory. A Ge on KBr substrate beamsplitter with a liquid nitrogen-cooled wide band mercury cadmium telluride (MCT) detector for the mid-IR interval (400-4000 cm^{-1}), and a Si beamsplitter

with a room temperature deuterated L-alanine doped triglycine sulfate (DLaTGS) detector with a polyethylene window for the far-IR range (50-600 cm^{-1}), were used. Each spectrum was the sum of 128 scans, at 2 cm^{-1} resolution, and the 3-term Blackman–Harris apodization function was applied. Under these conditions, the wavenumber accuracy was better than 1 cm^{-1}. The spectra were corrected for the frequency dependence of the penetration depth of the electric field in ATR (considering a mean reflection index of 1.25) using the Opus 7.2 spectroscopy software.

H_2O-DNA$_{hyd}$, D_2O-DNA$_{hyd}$ (with and without drug) and DNA$_{lyoph}$ samples were measured.

MicroRaman Measurements

The Raman microspectroscopy data were obtained at the QFM-UC (Coimbra, Portugal). The spectra were recorded in the 100-3750 cm^{-1} range, in a WITec confocal Raman microscope system alpha300 R, coupled to an Ultra-High-Throughput-Spectrometer UHTS 300 VIS-NIR (300 mm focal length; 600 lines/mm blazed for 500 nm grating). The detection system was a thermoelectrically cooled CCD camera with Peltier cooling down to -55 °C, chip with 1650x200 pixels, front-illuminated with NIR/VIS AR coating, spectral resolution <0.8 cm^{-1}/pixel. The 532 nm line of a diode laser was used as the excitation radiation, yielding *ca.* 30 mW at the sample position. An objective Zeiss Epiplan 100x/0.8 (NA 0.80) was used. 10 spectra were collected *per* sample, with 5 accumulations and 10 s of exposure time.

H_2O-DNA$_{hyd}$, D_2O-DNA$_{hyd}$ (with and without drug) and DNA$_{lyoph}$ samples were measured.

Data Analysis

INS data was converted to the conventional scattering law, S(Q, ν) *vs* energy transfer (in cm^{-1}) using the MANTID program (version 3.4.0) [8]]. The spectrum of the empty Al can was subtracted from the measured data for all DNA samples, with a view to better identify the low frequency vibrational features of the nucleic acid.

QENS data was reduced from raw data into energy transfer spectra using the program DAVE (version 2.6, developed at the National Institute of Standards and Technology (NIST) Center for Neutron Research, Gaithersburg, Maryland, USA [9]). QENS spectra were corrected for detector efficiency. Data at low temperature (<10 K) was used as the resolution functions.

Fitting of the QENS spectra was performed with DAVE. The systems were accurately represented by applying one Dirac Delta function (elastic component), and two Lorentzians (quasielastic contributions) characterising the slow motions of DNA´s backbone (Γ_{global}, narrow Lorentzian) and the faster dynamics of the water molecules within DNA´s spine of hydration (Γ_{local}, broader Lorentzian), equation (3) becoming,

$$S_{inc}(Q,\omega) = \exp(-Q^2\langle u^2\rangle)\left[A_0(Q)\delta(\omega) + \left(1 - A_0(Q)\right)L_{local}(Q,\omega)\right]\otimes L_{global}(Q,\omega)\otimes RF(Q) \qquad (6)$$

(where RF represents the instrument resolution function). A small intensity sloping background was added, for better fitting.

FWHM values were extracted from each of the Lorentzian functions, and the translational diffusion coefficients (D_T) and reorientation times (τ_T^{jump}, the mean residence time of a water molecule in each possible location) (at temperature T) were obtained according to a non-diffusive jump reorientation model [10-12] that follows the equation:

$$\Gamma_T(Q) = \frac{D_T Q^2}{1 + D_T Q^2 \tau_T^{jump}} \qquad (7)$$

Figures

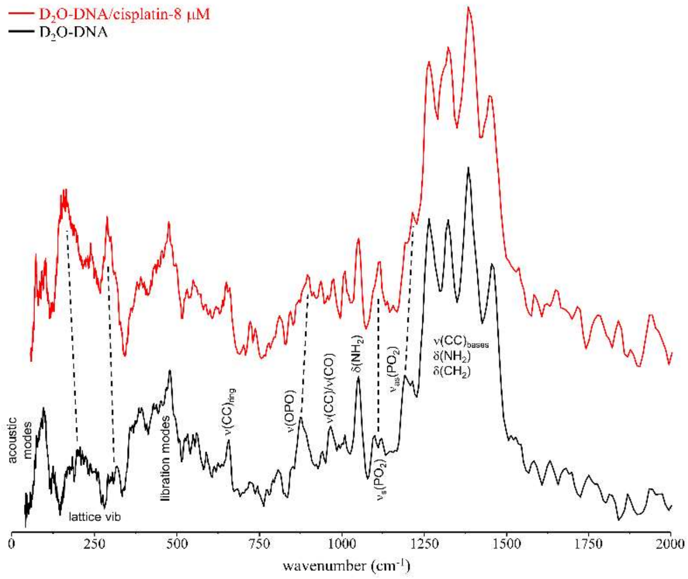

Figure S1 – INS spectra (at 10 K) of D_2O-DNA$_{hyd}$ untreated and upon incubation (for 48 h) with cisplatin-8 μM.
(The main drug-triggered vibrational changes in DNA are shown by dashed lines).

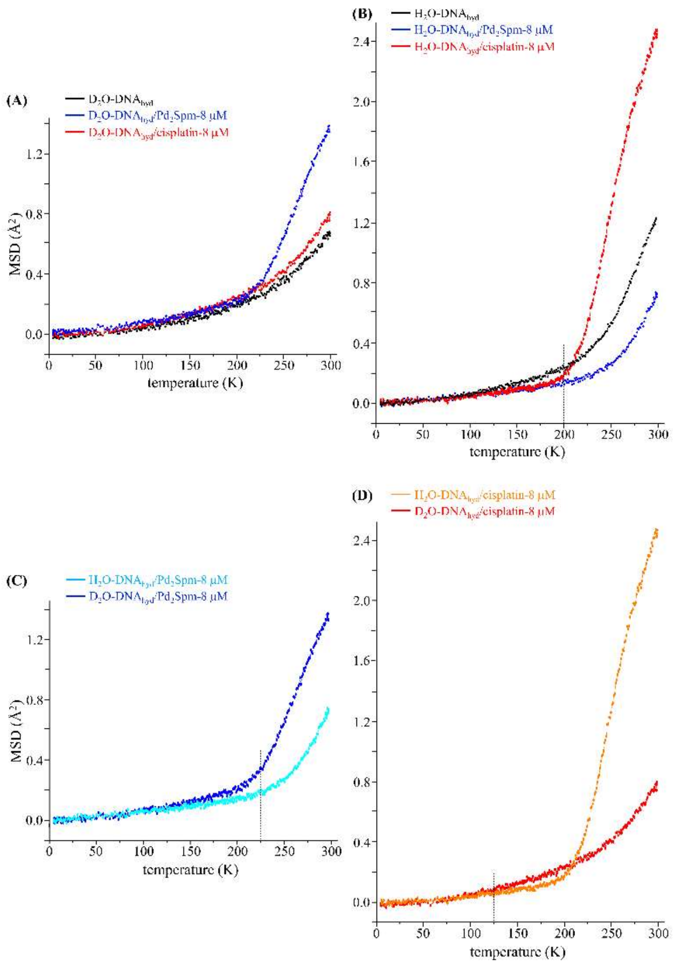

Figure S2 – Temperature variation of the mean-squared displacements for untreated and drug-exposed (8 μM) D_2O- and H_2O-DNA$_{hyd}$.

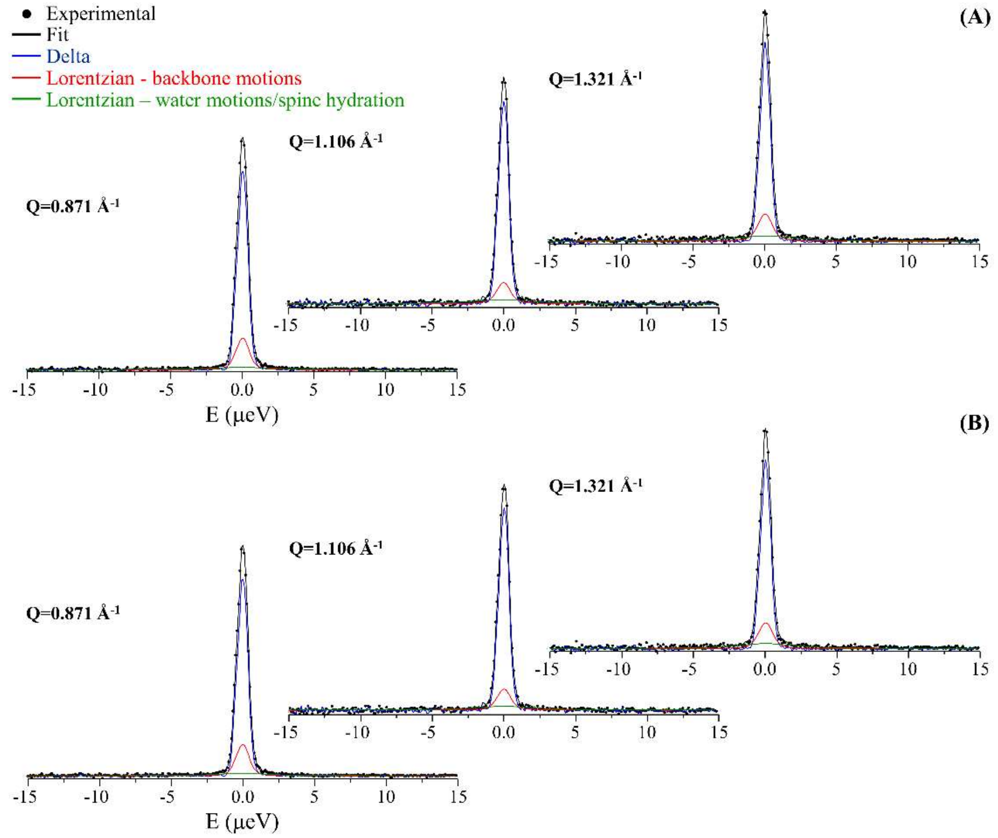

Figure S3 – QENS spectra (298 K) for H_2O-DNA_{hyd} – untreated (A) and Pd_2Spm-treated (8 μM) (B) – fitted using two Lorentzians and one Delta functions, at some typical Q values.

References

1. Codina, G.; Vidal, R.; Martin-Casabona, N.; Miravitlles, M.; Martin, C., Multidrug-resistant tuberculosis caused by 'W'-related strains in three immunocompetent foreign-born patients. *Int J Tuberc Lung Dis* **1999,** 3, (1), 82-4.
2. Fiuza, S. M.; Amado, A. M.; Parker, S. F.; Marques, M. P. M.; Batista de Carvalho, L. A. E., Conformational insights and vibrational study of a promising anticancer agent: the role of the ligand in Pd(II)-amine complexes. *New J Chem* **2015,** 39, (8), 6274-6283.
3. Parker, S. F.; Lennon, D.; Albers, P. W., Vibrational Spectroscopy with Neutrons: A Review of New Directions. *Appl Spectrosc* **2011,** 65, (12), 1325-1341.
4. Parker, S. F.; Fernandez-Alonso, F.; Ramirez-Cuesta, A. J.; Tomkinson, J.; Rudić, S.; Pinna, R. S.; Gorini, G.; Castanon, J. F., Recent and future developments on TOSCA at ISIS. *J Phys Conf Ser* **2014,** 554.
5. Pinna, R. S.; Rudić, S.; Capstick, M. J.; McPhail, D. J.; Pooley, D. E.; Howells, G. D.; Gorini, G.; Fernandez-Alonso, F., Detailed characterisation of the incident neutron beam on the TOSCA spectrometer. *Nucl Instrum Meth A* **2017,** 870, 79-83.
6. Pinna, R. S.; Rudić, S.; Parker, S. F.; Armstrong, J.; Zanetti, M.; Skoro, G.; Waller, S. P.; Zacek, D.; Smith, C. A.; Capstick, M. J.; McPhail, D. J.; Pooley, D. E.; Howells, G. D.; Gorini, G.; Fernandez-Alonso, F., The neutron guide upgrade of the TOSCA spectrometer. *Nucl Instrum Meth A* **2018,** 896, 68-74.
7. Meyer, A.; Dimeo, R. M.; Gehring, P. M.; Neumann, D. A., The high-flux backscattering spectrometer at the NIST Center for Neutron Research. *Rev Sci Instrum* **2003,** 74, (5), 2759-2777.
8. Arnold, O.; Bilheux, J. C.; Borreguero, J. M.; Buts, A.; Campbell, S. I.; Chapon, L.; Doucet, M.; Draper, N.; Leal, R. F.; Gigg, M. A.; Lynch, V. E.; Markvardsen, A.; Mikkelson, D. J.; Mikkelson, R. L.; Miller, R.; Palmen, K.; Parker, P.; Passos, G.; Perring, T. G.; Peterson, P. F.; Ren, S.; Reuter, M. A.; Savici, A. T.; Taylor, J. W.; Taylor, R. J.; Tolchenoy, R.; Zhou, W.; Zikoysky, J., Mantid-Data analysis and visualization package for neutron scattering and mu SR experiments. *Nucl Instrum Meth A* **2014,** 764, 156-166.
9. Azuah, R. T.; Kneller, L. R.; Qiu, Y. M.; Tregenna-Piggott, P. L. W.; Brown, C. M.; Copley, J. R. D.; Dimeo, R. M., DAVE: A Comprehensive Software Suite for the Reduction, Visualization, and Analysis of Low Energy Neutron Spectroscopic Data. *J Res Natl Inst Stan* **2009,** 114, (6), 341-358.
10. Laage, D.; Hynes, J. T., A molecular jump mechanism of water reorientation. *Science* **2006,** 311, (5762), 832-835.
11. Laage, D., Reinterpretation of the Liquid Water Quasi-Elastic Neutron Scattering Spectra Based on a Nondiffusive Jump Reorientation Mechanism. *J Phys Chem B* **2009,** 113, (9), 2684-2687.
12. Laage, D.; Elsaesser, T.; Hynes, J. T., Water Dynamics in the Hydration Shells of Biomolecules. *Chem Rev* **2017,** 117, (16), 10694-10725.
